# Evaluation of an amino acid residue critical for the specificity and activity of human Gb3/CD77 synthase

**DOI:** 10.1007/s10719-016-9716-9

**Published:** 2016-08-18

**Authors:** Radoslaw Kaczmarek, Katarzyna Mikolajewicz, Katarzyna Szymczak, Maria Duk, Edyta Majorczyk, Anna Krop-Watorek, Anna Buczkowska, Marcin Czerwinski

**Affiliations:** 1 Laboratory of Glycoconjugate Immunochemistry, Hirszfeld Institute of Immunology and Experimental Therapy, Polish Academy of Sciences, Wroclaw, Poland; 2 Confocal Microscopy Laboratory, Wroclaw Research Centre EIT+, Wroclaw, Poland; 3 Institute of Physiotherapy, Faculty of Physiotherapy and Physical Education, Opole University of Technology, Opole, Poland; 4 Department of Biotechnology and Molecular Biology, University of Opole, Opole, Poland; 5 Institute of Biochemistry and Biophysics, Polish Academy of Sciences, Warsaw, Poland

**Keywords:** Gb3/CD77 synthase, P1PK blood group system, NOR polyagglutination, Glycopshingolipids, Site-directed mutagenesis

## Abstract

Human Gb3/CD77 synthase (α1,4-galactosyltransferase) is the only known glycosyltransferase that changes acceptor specificity because of a point mutation. The enzyme, encoded by *A4GALT* locus, is responsible for biosynthesis of Gal(α1–4)Gal moiety in Gb3 (CD77, P^k^ antigen) and P1 glycosphingolipids. We showed before that a single nucleotide substitution c.631C > G in the open reading frame of *A4GALT*, resulting in replacement of glutamine with glutamic acid at position 211 (substitution p. Q211E), broadens the enzyme acceptor specificity, so it can not only attach galactose to another galactose but also to *N*-acetylgalactosamine. The latter reaction leads to synthesis of NOR antigens, which are glycosphingolipids with terminal Gal(α1–4)GalNAc sequence, never before described in mammals. Because of the apparent importance of position 211 for enzyme activity, we stably transfected the 2102Ep cells with vectors encoding Gb3/CD77 synthase with glutamine substituted by aspartic acid or asparagine, and evaluated the cells by quantitative flow cytometry, high-performance thin-layer chromatography and real-time PCR. We found that cells transfected with vectors encoding Gb3/CD77 synthase with substitutions p. Q211D or p. Q211N did not express P^k^, P1 and NOR antigens, suggesting complete loss of enzymatic activity. Thus, amino acid residue at position 211 of Gb3/CD77 synthase is critical for specificity and activity of the enzyme involved in formation of P^k^, P1 and NOR antigens. Altogether, this approach affords a new insight into the mechanism of action of the human Gb3/CD77 synthase.

## Introduction

Glycosyltransferases are a large group of enzymes that synthesize carbohydrate moieties of glycoproteins, glycosaminoglycans and glycosphingolipids. These molecules are involved in many processes, such as cell differentiation, signal transduction, immune response and pathogen infection [1]. They also contribute to diversity of many human blood group antigens belonging to blood group systems such as ABO, Lewis or P1PK [2, 3]. The reactions catalyzed by glycosyltransferases involve transferring sugar residues from the sugar donors to the acceptor molecules, forming glycosidic bonds. Donor sugar substrates are usually activated in the form of nucleoside diphosphate sugars (*e.g.* UDP-Gal) or nucleoside monophosphate sugars (*e.g.* CMP-NeuNAc). The acceptor substrates can be sugars, lipids, proteins or small molecules such as coumarin [1, 4]. Many glycosyltransferases (nearly all belonging to the GTA superfamily) require divalent metal ion (usually manganese) at the catalytic center, where it is coordinated by two D residues that form the DXD motif [4]. In general, glycosyltransferases reveal high donor and acceptor specificity, and it was demonstrated that mutations in genes encoding glycosyltransferases may lead to changes in either [5]. However, while change in donor specificity is a well described phenomenon and has been shown for several enzymes, such as ABO transferase [6] or β1,4-galactosyltransferase [7], the change of acceptor specificity has been demonstrated for only one enzyme, Gb3/CD77 synthase, which is a glycosphingolipid-specific glycosyltransferase [8].

Glycosphingolipids are amphipathic compounds consisting of hydrophilic carbohydrate and hydrophobic ceramide moieties [9]. Glycosphingolipids constitute a significant portion of mammalian cell membranes, including intracellular compartments. In humans, four major types of glycosphingolipid neutral root structures (called series) can be distinguished: the globo (GalNAcβ1-3Galα1-4Galβ1-4Glc), lacto (Galβ1-3GlcNAcβ1-3Galβ1-4Glc), neolacto (Galβ1-4GlcNAcβ1-3Galβ1-4Glc) and ganglio (Galβ1-3GalNAcβ1-4Galβ1-4Glc) [10, 11]. In addition, glycosphingolipids of all series may contain sialic acid and these are traditionally (albeit confusingly) called gangliosides or acidic glycosphingolipids; most of them have ganglio or neolacto core chains. Glycosphingolipids on blood and tissue cells may carry histo-blood group antigens, such as A, B, P^k^ or P1 [3].

**Fig. 1:**
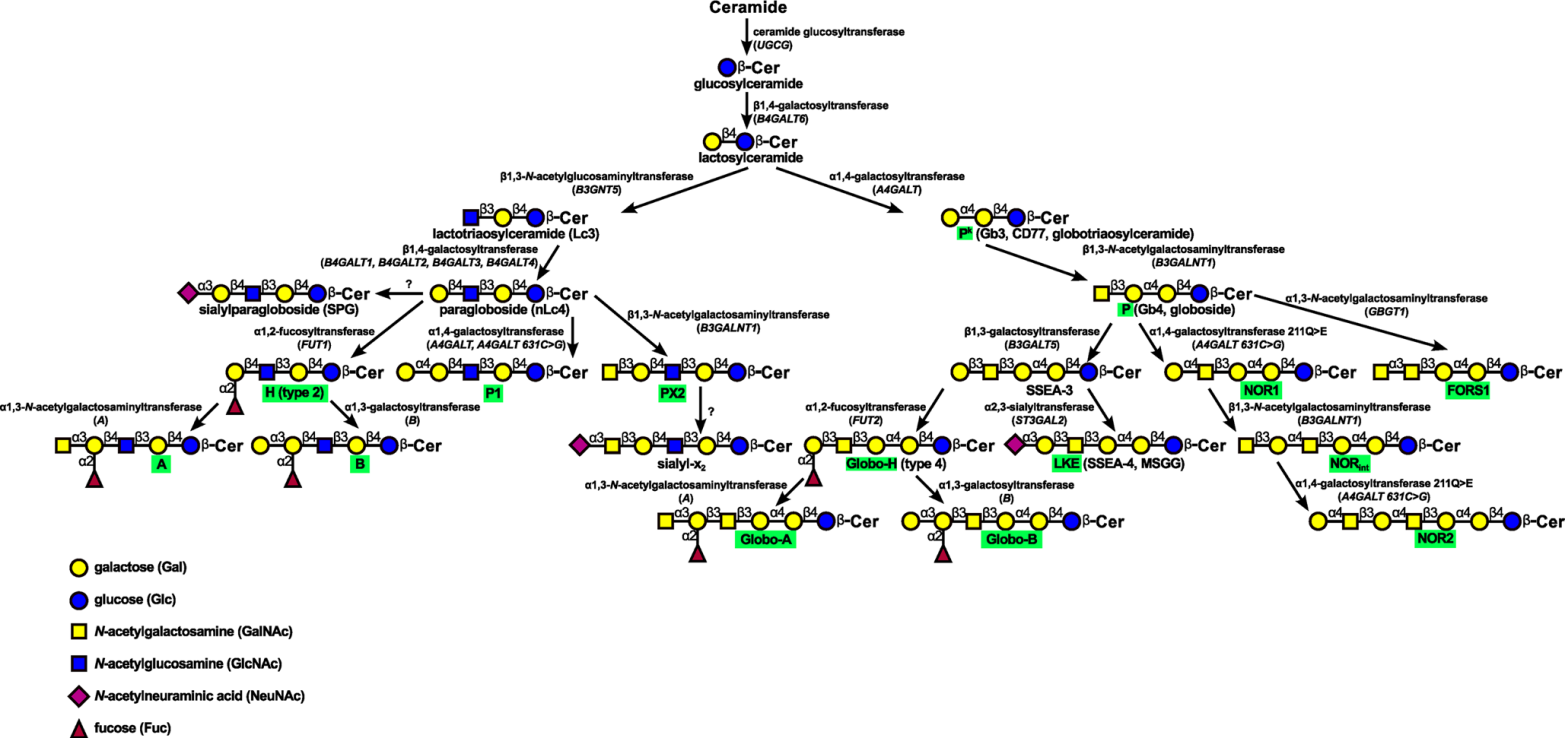
Schematic representation of biosynthesis of ABO, P1PK, GLOB and FORS blood group antigens

Gb3/CD77 synthase (UDP-Gal:lactosylceramide α1,4-galactosyltransferase; α1,4-galactosyltransferase), encoded by *A4GALT* gene, catalyzes the transfer of galactose from UDP-galactose to lactosylceramide (LacCer), giving rise to globo-series pathway. The product is called globotriaosylceramide (Gb3), CD77 or P^k^ blood group antigen [12]. P1 antigen is synthesized further downstream from lactosylceramide in the neolacto-series pathway, which is a separate entity. Paragloboside, the precursor for P1 antigen, serves also as a precursor for human histo-blood group H, A and B antigens (Fig. 1). Recently, we have shown that Gb3/CD77 synthase is responsible for synthesis of P1 blood group antigen [13]. Both P^k^ and P1 antigens are terminated with Gal(α1–4)Gal moiety. P^k^ antigen can be elongated by β1,3-*N*-acetylgalactosaminyltransferase (P synthase) giving rise to globoside (P antigen, Gb4), which is the most abundant neutral glycosphingolipid on erythrocytes [14]. The same enzyme synthesizes PX2 glycosphingolipid from paragloboside [15] (Fig. 1). Both antigens are now classified as members of the GLOB blood group system [16]. The Forssman antigen, very rare in humans but present in several mammalian species, is synthesized when a single point mutation is present in *GBGT1* (otherwise a pseudogene in humans) encoding α1,3-*N*-acetylgalactosaminyltransferase [17]. The Forssman antigen is now classified as a sole member of the FORS blood group system [16].

Both P^k^ and P1 antigens belong to the P1PK blood group system, and the presence or absence of P1 antigen on erythrocytes determines the P_1_ (P1-positive) or P_2_ (P1-negative) blood group, respectively [3]. The null phenotype caused by mutation in *A4GALT* gene, is called p [3]. Despite several attempts, the molecular background of the P1PK blood group system is still not fully elucidated. Several authors have shown that the expression levels of *A4GALT* mRNA is higher in P_1_ than in P_2_, and there is a general agreement that the upregulated transcript may cause increased production of Gb3/CD77 synthase [18–20]. However, despite finding several SNPs associated with P_1_/P_2_ status, no credible mechanism for allelic variation in *A4GALT* gene expression has been proposed.

The NOR antigen, fully elucidated in our laboratory, is an unusual glycosphingolipid with terminal Gal(α1–4)GalNAc moiety, found in erythrocytes of individuals with the rare NOR polyagglutination syndrome [21]. The erythrocytes of NOR-positive individuals contain unique neutral glycosphingolipids formed by the elongation of globoside: NOR1, Gal(α1–4)GalNAc(β1–3)Gal(α1–4)Gal(β1–4)GlcCer; NORint, GalNAc(β1–3)Gal(α1–4)GalNAc(β1–3)Gal(α1–4)Gal(β1–4)GlcCer; and NOR2, Gal(α1–4)GalNAc(β1–3)Gal(α1–4)GalNAc(β1–3)Gal(α1–4)Gal(β1–4)GlcCer [22]. We demonstrated that a single point mutation c.631C > G in *A4GALT* resulting in replacement of glutamine with glutamic acid at position 211 (substitution p. Q211E) broadens the acceptor specificity of the Gb3/CD77 synthase; as a result, the variant enzyme is able to catalyze the synthesis of two different terminal disaccharide moieties: Gal(α1–4)Gal (in P^k^ and P1 antigens) and Gal(α1–4)GalNAc (in NOR antigens) [8] (Fig. 1). The NOR antigen has been classified as the third member of the P1PK blood group system [16]. The NOR phenotype is rare, but its biological role is significant, because natural anti-NOR antibodies present in human sera recognize the terminal trisaccharide unit (Gal(α1–4)GalNAc(β1–3)Gal) of NOR1 and NOR2 glycosphingolipids [23]. The presence of these antibodies, common in general population, underlies a rare phenomenon known as inheritable NOR polyagglutination: red blood cells of NOR-positive individuals are agglutinated by most human sera, which disqualifies such individuals as blood donors [24].

Gb3/CD77 synthase is the first described enzyme in which a single amino acid substitution leads to the change of acceptor specificity, and this finding suggests that amino acid residue 2011 determines the catalytic properties of the Gb3/CD77 synthase. Here we use site-directed mutagenesis combined with quantitative analysis of glycosphingolipid antigens expression to evaluate the role of amino acid residue 211 in the specificity and activity of the enzyme.

## Materials and methods

### Site-directed mutagenesis

Site-directed mutagenesis was performed using overlap-extension PCR, as described previously [8]. In the first PCR reaction, two fragments of *A4GALT* were created, each containing the overlapping site with introduced mutation. In the second reaction, the PCR products were duplexed to generate new template DNA. During the overlap extension phase, each fused product was amplified using primers complementary to the pCAG vector (pCAGsense and pCAGanti). The resulting full-length gene fragments were directly ligated into the pGEM-T Easy Vector and then digested with XhoI and NotI (Fermentas, Vilnius, Lithuania), cloned into appropriately digested pCAG vector and confirmed by sequencing (Genomed, Warsaw, Poland) using primers PkSeqFor and PkSeqRev. The plasmids were purified using maxi prep kit (Qiagen, Venlo, Netherlands) according to the manufacturer’s instruction. The reaction was performed in a MJ Mini gradient PCR apparatus (BioRad, Hercules, CA, USA). 20 μl reaction mixture contained: amount of DNA solution containing approximately 200 ng of the template, 0.2 mM forward and reverse primers, 0.2 mM dNTPs, 1.5 mM MgCl_2_, HF polymerase buffer (1:10 dilution), 1 unit Phusion High-Fidelity DNA Polymerase (Fermentas, Vilnius, Lithuania). The DNA fragments were purified with gel extraction kit (Gel-Out, A&A Biotechnology, Gdynia, Poland). The sequences of primers are shown in Table 1, and the conditions of PCR reactions are shown in Table 2.

**Table 1:** Nucleotide sequences of primers used in site-directed mutagenesis and sequencing

Name of primer	Sequence (5′ → 3′)
PkmutDsense	TGCTGGGCACCGACTCCCGCTAC
PkmutDanti	GTAGCGGGAGTCGGTGCCCAGCA
PkmutNsense	TGCTGGGCACCAACTCCCGCTAC
PkmutNanti	GTAGCGGGAGTTGGTGCCCAGCA
pCAGsense	CGTGCTGGTTGTTGTGCTGTCTCA
pCAGanti	ACAAACGCACACCGGCCTTATTCC
PkSeqFor	TCGCACTCATGTGGAAG
PkSeqRev	AGTACATTTTCATGGCCT

**Table 2:** PCR conditions used in site-directed mutagenesis

	Mutagenesis – first step			Mutagenesis – second step		
	temp [°C]	time [s]	cycle	temp [°C]	time [s]	cycle
Initial denaturation	94	180	1	94	180	1
Denaturation	94	30	30	94	30	30
Annealing	65–75	30	30	65–75	30	30
Extension	72	75	30	72	100	30
Final extension	72	600	1	72	600	1

### Cell culture and transfection

The teratocarcinoma cell line 2102Ep was a generous gift from Dr. Peter W. Andrews (University of Sheffield, UK) [25]. The cells were grown in Dulbecco’s Modified Eagle Medium (DMEM) containing 4.5 g/l glucose (Invitrogen, Carlsbad, CA, USA), 10 % fetal calf serum (Invitrogen, Carlsbad, CA, USA), and 2 mM GlutaMAX (Invitrogen, Carlsbad, CA, USA) under a humidified atmosphere of 5 % CO_2_ and at 37 °C. Culture medium was changed every second or third day, and after reaching 85–90 % confluence, the cells were passaged using 0.25 % trypsin/1 mM EDTA.

The cells were seeded at 2 × 10^5^ cells per well in six-well plates the day before transfection, so that confluence at day of transfection was about 60 %. 3 h before transfection the medium was replaced with fresh DMEM. The cells were transfected using 10 μg polyethylenimine (Polysciences, Warrington, PA, USA). Plasmid DNA in an amount of 3 μg was diluted in 200 μl 0.15 M NaCl and then mixed briefly with polyethylenimine. The transfection mixture was incubated for 20 min at room temperature and then added dropwise to each well. The next day (after 18–20 h) medium was replaced with fresh DMEM. 48 h after transfection, cells were subjected to puromycin selection (Sigma-Aldrich, St. Louis, MO, USA) at a concentration of 0.44 μg/ml. The medium with antibiotic was changed daily for 10 days and then every 2 days. Selection was carried out until the non-transfected control cells were dead.

### Antibodies

The mouse monoclonal anti-NOR antibody, nor118 was obtained in our laboratory before and used as a diluted culture supernatant [24]. Antibodies: human anti-P1, mouse anti-P1, goat anti-mouse IgM conjugated with fluorescein isothiocyanate (FITC), and goat anti-mouse IgG conjugated with FITC antibodies were purchased from Immucor Inc. (Norcross, GA, USA), CE-Immundiagnostika (Eschelbronn, Germany), Santa Cruz Biotechnology (Dallas, TX, USA) and Dako (Glostrup, Denmark), respectively. The goat anti-human IgM conjugated with FITC antibodies was purchased from Pierce (Rockford, IL, USA), and biotinylated anti-mouse antibody and biotinylated anti-human antibody were purchased from Sigma-Aldrich (St. Louis, MO, USA).

### Flow cytometry

The cells were incubated with 100 μl appropriately diluted primary antibodies (human anti-P1 1:10, mouse anti-P1 1:10, anti-NOR 1:20) for 60 min on ice. Then the cells were washed (all washes and dilutions were done with PBS) and incubated with 100 μl (diluted 1:50) FITC-labeled anti-mouse IgM antibody for 40 min on ice in the dark. The cells were washed and approximately 5 × 10^5^ cells were suspended in 750 μl of cold PBS and analyzed by flow cytometry using FACSCalibur (BD Biosciences, Franklin Lakes, NJ, USA). The number of events analyzed was 10,000/gated cell population. The analysis of the results was carried out using Flow Jo software (Tree Star, Ashland, OR, USA).

### Flow cytometric quantification of cell surface receptor expression

The cells were prepared as described in the Flow Cytometry section. The QIFIKIT (Dako, Glostrup, Denmark) bead populations with defined quantities of antibody molecules attached per a single bead were incubated with FITC-labeled secondary antibody (goat anti-mouse IgG conjugated with FITC included in the QIFIKIT or goat anti-mouse IgM, in which case the antibody binding capacities were corrected for differences in fluorophore to protein ratios). The calibration beads were then analyzed by flow cytometry and the data were used for plotting the calibration curves (mean fluorescence intensity versus antibody binding capacity). The cells were then analyzed by flow cytometry and the antigen density was calculated by interpolation from the calibration curve as described in the manufacturer’s protocol. The cells stained with the respective control were analyzed using the same protocol.

### Extraction and purification of glycosphingolipids

The isolation and fractionation of glycosphingolipids and the orcinol staining were performed as described previously [22]. Cellular lipids were extracted with chloroform/methanol from 10^7^ 2102Ep cells. The neutral glycosphingolipids were separated from the phospholipids and gangliosides, purified in peracetylated form, de-*O*-acetylated, and desalted. Glycosphingolipid samples were solubilized in chloroform/methanol (2:1, *v*/v), applied to HPTLC plates (Kieselgel 60, Merck, Darmstadt, Germany), and developed with chloroform/methanol/water (55:45:9, *v*/v/v). The dried plates were immersed in 0.05 % polyisobutylmethacrylate (Aldrich, Steinheim, Germany) in hexane for 1 min, dried, sprayed with TBS (0.05 M Tris buffer, 0.15 M NaCl (pH 7.4)), and blocked in 5 % BSA. For antibody assays, the plates were successively overlaid with 1) primary antibody diluted in TBS/1 % BSA (TBS-BSA) for 1–1.5 h; 2) biotinylated goat anti-mouse Ig antibody (Dako, Glostrup, Denmark), diluted 1:5000 with TBS-BSA; 3) ExtrAvidin-alkaline phosphatase conjugate (Sigma-Aldrich, St. Louis, MO, USA) diluted 1:1000 with TBS/ BSA/0.2 % Tween20 for 1 h; and 4) the substrate solution (nitro blue tetrazolium/5-bromo-4-chloro-3-indolyl phosphate, Sigma-Aldrich). Other details were as described previously [22, 26]. Each HPTLC experiment was repeated three times.

### Quantitative analysis of transcripts

Total RNA from different 2102Ep clones was prepared using RNeasy mini kit (Qiagen, Venlo, Netherlands) and the complementary DNAs (cDNAs) were synthesized using SuperScript III First-Strand Synthesis kit (Life Technologies, Carlsbad, CA, USA) with oligo(dT) primers. Quantitative polymerase chain reaction (qPCR) was performed on 30 ng of cDNA using the 7500 Fast Real-Time PCR System (Life Technologies), according to the manufacturer’s instruction. The RNA samples not treated with reverse transcriptase (RT-) were also evaluated to check for genomic DNA contamination. The *A4GALT* transcripts were detected with Custom TaqMan Gene Expression Assay. The ORF sequences were chosen in assay design to enable detection of transcripts originating from plasmids. A predesigned TaqMan assay targeting exon 2–3 boundary (Hs00213726_m1; Life Technologies) was also used to ensure equal amount of the endogenous *A4GALT* transcript in untransfected and transfected cells. The transcript quantities were normalized to *ACTB* (β-actin) endogenous control (assay Hs99999903_m1). All samples were run in triplicates. The untransfected cells sample (NAT) was used as the calibrator. Data were analyzed using Sequence Detection software Version 1.3.1 (Life Technologies). Target nucleotide sequences are shown in Table 3, while real-time PCR conditions are in Table 4.

**Table 3:** Target nucleotide sequences within *A4GALT* open reading frame used for design of Custom TaqMan Gene Expression Assay

Name of target sequence	Sequence (5′ → 3′)
A4gf	CTGCACCCT
A4gr	TTCTCAAGAAC

**Table 4:** Real-time PCR conditions used for quantitative analysis of *A4GALT* transcripts

Real-time PCR System	Reaction format	Reaction volume	Thermal cycling conditions			
			Parameter	Initial denaturation	PCR (40 cycles)	
					Denaturation	Annealing/Extension
			Temperature (°C)	95	95	60
7500 Fast	96-well plate	20 μl	Time (mm:ss)	10:00	0:15	1:00

## Results

**Fig. 2:**
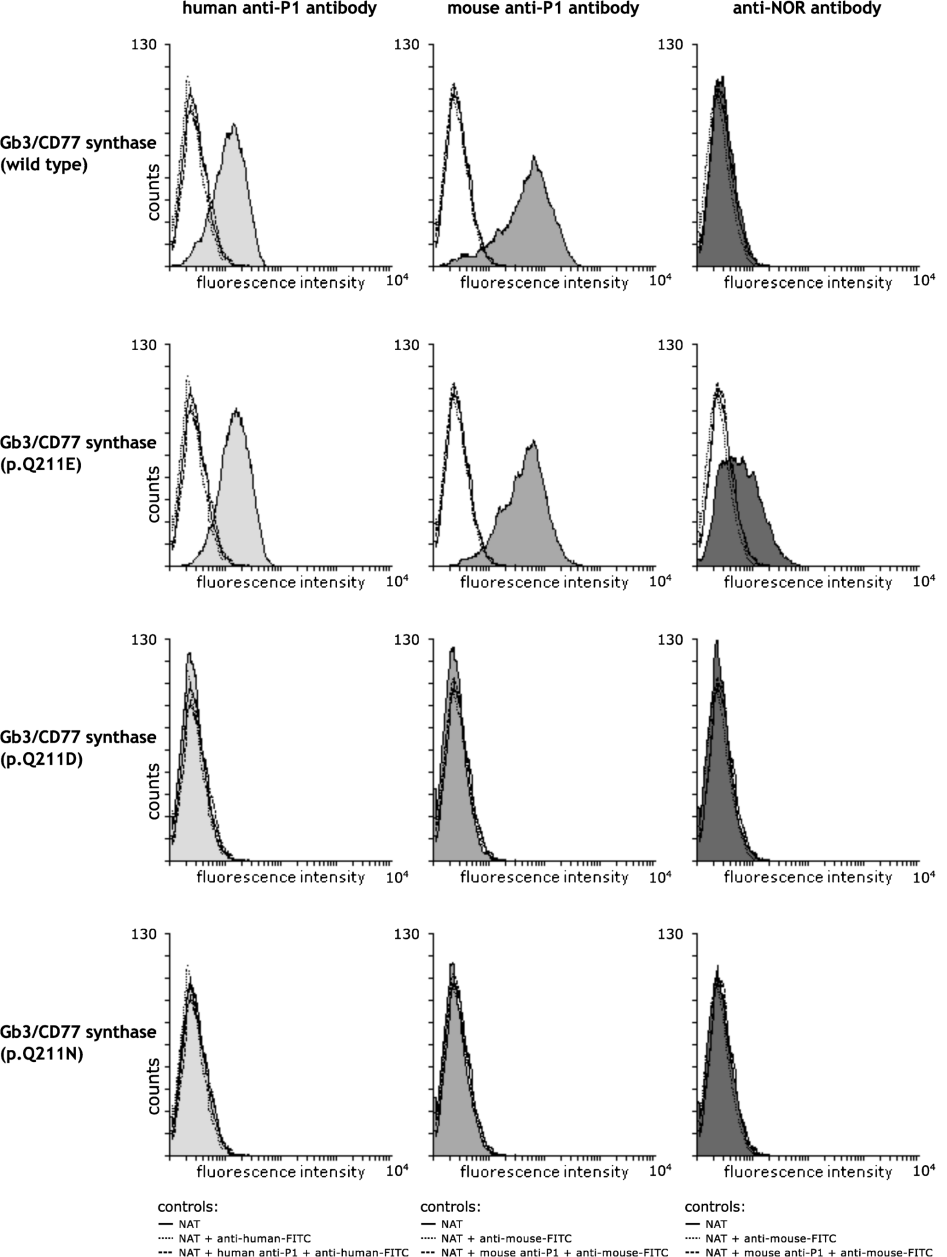
Flow cytometry analysis of the binding of human anti-P1, mouse anti-P1 and anti-NOR antibodies to 2102Ep cells transfected with vectors encoding various forms of Gb3/CD77 synthase: the consensus Gb3/CD77 synthase (wild type) containing a Q residue at position 211, Gb3/CD77 synthase p. Q211E (E at position 211), Gb3/CD77 synthase p. Q211D (D at position 211), Gb3/CD77 synthase p. Q211N (N at position 211)

We showed before that c.631C > G mutation in *A4GALT* resulting in substitution of glutamine with glutamic acid at position 211 (substitution p. Q211E) of the Gb3/CD77 synthase is responsible for synthesis of NOR antigen [8]. To examine how the substitution of 211Q by other amino acid residues influences synthesis of P^k^, P1 and NOR antigens, we stably transfected human teratocarcinoma 2102Ep cells with pCAG vectors encoding either the consensus Gb3/CD77 synthase or the enzyme with p. Q211N or p. Q211D substitutions. The results were evaluated by flow cytometry using the following antibodies: anti-NOR, human anti-P1 (which reacts with P1 antigen only) and mouse anti-P1 (which reacts with both P1 and P^k^ antigens). We found that cells transfected with the vector encoding Gb3/CD77 synthase with substitutions p. Q211N or p. Q211D did not express P^k^, P1 or NOR antigens (Fig. 2). In contrast, as before, cells transfected with the vector encoding consensus Gb3/CD77 synthase expressed P1 antigen, and cells transfected with the vector encoding Gb3/CD77 synthase with the previously described p. Q211E substitution expressed all three antigens: P^k^, P1 and NOR.

**Fig. 3:**
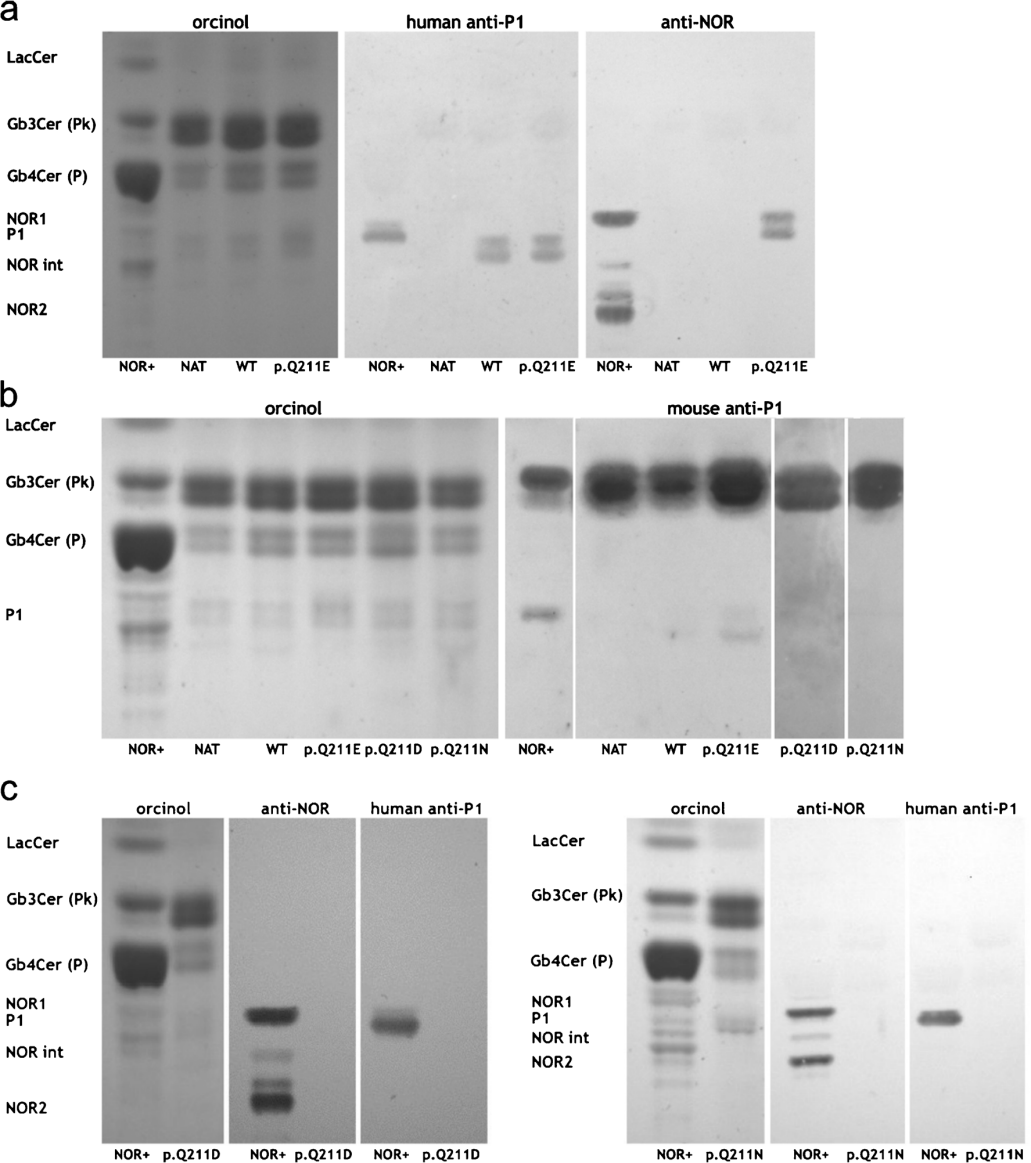
HPTLC analysis of neutral glycosphingolipids extracted from 2102Ep cells. The samples of neutral glycosphingolipids obtained from NOR-positive red blood cells (NOR+) and 2102Ep cells: untransfected (NAT), transfected with vector encoding the consensus Gb3/CD77 synthase (WT) and transfected with vectors encoding Gb3/CD77 synthase with p. Q211E (p. Q211E), p. Q211D (p. Q211D) or p. Q211N (p. Q211N) substitutions were detected by orcinol staining (orcinol) or by overlaying with human anti-P1 antibody (human anti-P1 antibody, panels 3a and 3c), mouse anti-P1 antibody (mouse anti-P1 antibody, panel 3b) and mouse anti-NOR antibody (anti-NOR antibody, panels 3a and 3c)

Neutral glycosphingolipid fractions obtained from the 2102Ep cells transfected with vectors encoding enzymes with p. Q211D and p. Q211N substitutions were analyzed by thin-layer chromatography (Fig. 3). The orcinol staining showed that Gb4Cer is the major neutral glycosphingolipid of NOR-positive erythrocytes (NOR+); LacCer and Gb3Cer were also detected, albeit in lower quantity. In contrast, 2102Ep cells showed different staining profiles, with Gb3Cer being the predominant species, smaller amounts of Gb4Cer and traces of LacCer in the case of all Gb3/CD77 variants. Since HPTLC analysis was done on different occasions, there are three panels with orcinol staining as standards. We found that human anti-P1 antibody, which reacts only with P1 antigen, detected P1 antigen in glycosphingolipids from NOR-positive erythrocytes (NOR+) and from 2102Ep cells transfected with vectors encoding the consensus Gb3/CD77 synthase (WT) and p. Q211E Gb3/CD77 synthase (p. Q211E) (Fig. 3a and c). No bands were visible in fractions obtained from 2102Ep cells transfected with p. Q211D (p. Q211D) or p. Q211N (p. Q211N) enzyme (Fig. 3a and b). The mouse anti-P1 antibody, which recognizes both P^k^ and P1 antigens, detected P^k^ antigen in glycosphingolipids from erythrocytes, as well as in all fractions from untransfected (NAT) or transfected 2102Ep cells, while a weak double band (the doublet representing isoforms differing in length of fatty acid chain in the ceramide moiety) corresponding to P1 antigen was present only in glycosphingolipids obtained from erythrocytes and 2102Ep cells transfected with vectors encoding the consensus enzyme (WT) and the p. Q211E variant (p. Q211E) (Fig. 3b).

Mouse anti-NOR antibody, which recognizes glycosphingolipids that terminate with Gal(α1–4)GalNAc moiety detected two bands in glycosphingolipids from NOR-positive erythrocytes (NOR+), corresponding to NOR1 (upper band) and NOR2 (lower band). The antibody detected a band corresponding to NOR1 only in glycosphingolipids obtained from cells transfected with the vector encoding p. Q211E enzyme (p. Q211E) (Fig. 3a). No band was detected in the neutral glycosphingolipid fraction obtained from the cells transfected with vectors encoding Gb3/CD77 p. Q211D synthase (p. Q211D) or Gb3/CD77 p. Q211N synthase (p. Q211N) (Fig. 3b).

**Fig. 4:**
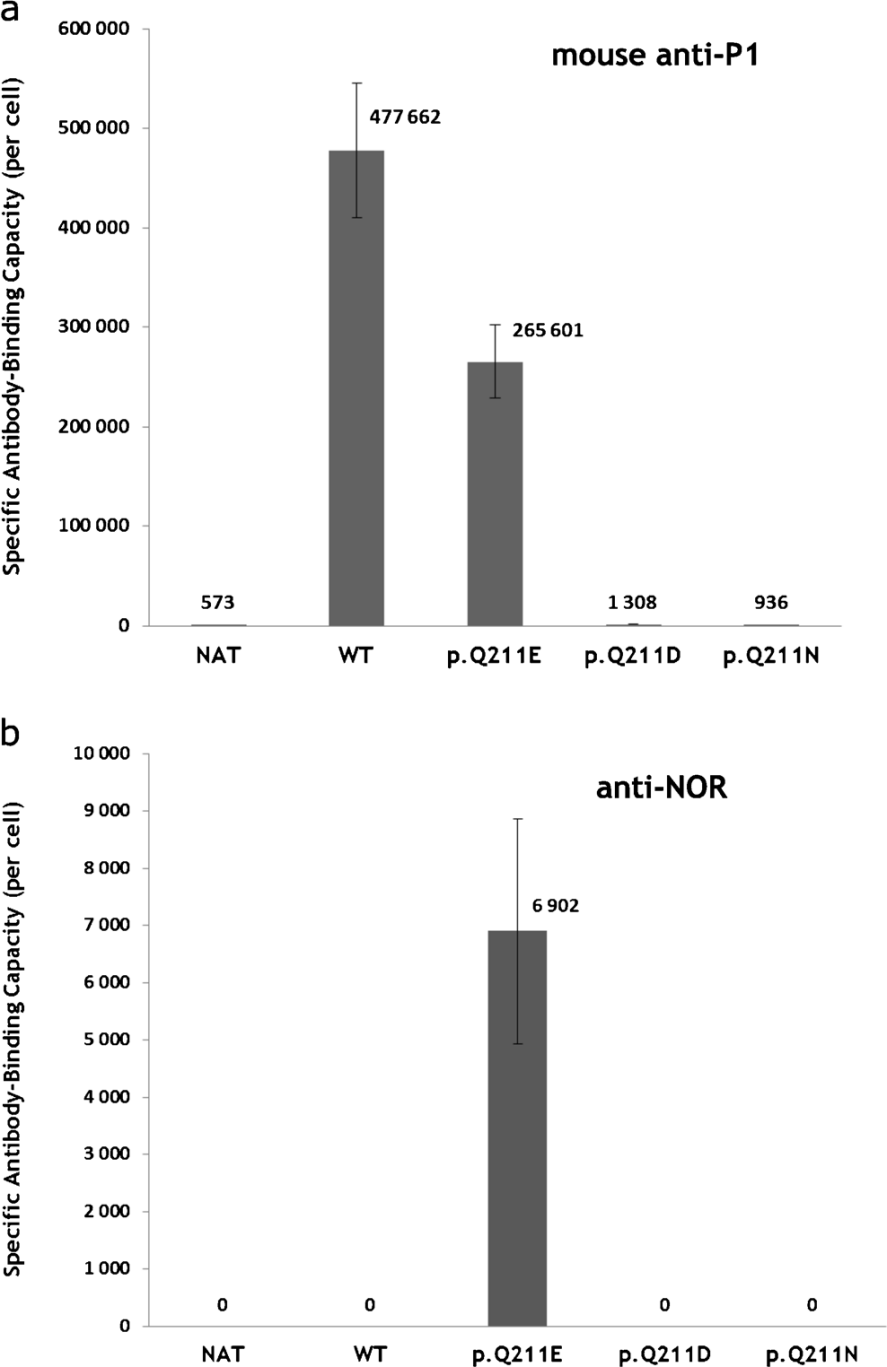
Flow cytometry quantification of specific antibody-binding capacity. Comparison of quantitative measurement of P1 and P^k^ antigens (**a**) and NOR antigens (**b**) on the surface of untransfected 2102Ep cells (NAT) and 2102Ep cells transfected with vectors encoding various forms of Gb3/CD77 synthase: consensus Gb3/CD77 synthase (WT); Gb3/CD77 synthase with E at position 211 (p. Q211E); Gb3/CD77 synthase with D at position 211 (p. Q211D) and Gb3/CD77 synthase with N at position 211 (p. Q211N)

To quantitatively evaluate antigen density on the cells transfected with vectors encoding various forms of Gb3/CD77 synthase we used QIFIKIT, which allowed to calculate the number of antibody molecules bound to cells using flow cytometry (Fig. 4). Since human anti-P1 antibody is not compatible with QIFIKIT (which employs mouse antibodies), we used mouse anti-P1 antibody to calculate specific antibody binding capacity on cells transfected with different vectors. We found that antibody binding capacity of the 2102Ep cells transfected with the vector encoding the consensus Gb3/CD77 synthase (WT) was smaller (265,601 ± 37,000) in comparison with the cells harboring the vector encoding the p. Q211E mutein (p. Q211E) (477,662 ± 67,000), which amounts to 55 % of binding capacity of the 2102Ep cells altered to produce the p. Q211E variant of the enzyme (Fig. 4a). In contrast, antibody binding capacity of the cells transfected with vectors encoding enzyme with p. Q211N (p. Q211N) or p. Q211D (p. Q211D) substitutions were much lower (1308 ± 176 and 936 ± 208, respectively), thus being close to the antibody binding capacity of untransfected cells (NAT), which was 573 ± 375. Anti-NOR antibody bound only to the cells transfected with the vector encoding Gb3/CD77 p. Q211E synthase (p. Q211E), showing antibody binding capacity of 6902 ± 1966 (Fig. 4b).

**Fig. 5:**
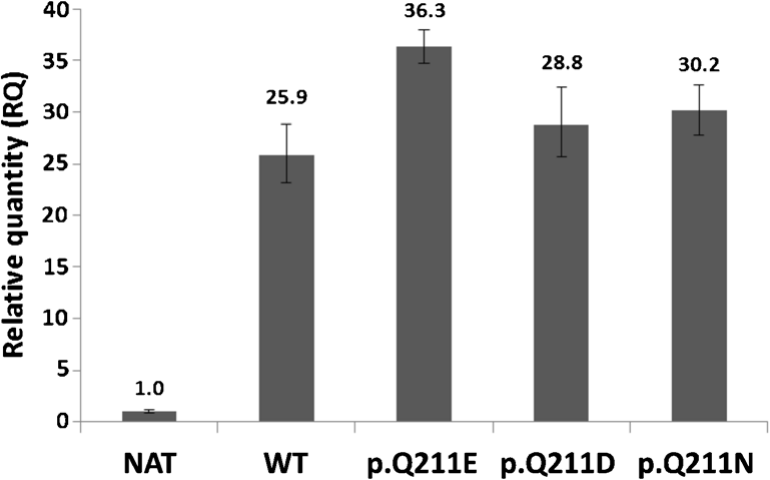
Quantitative analysis of *A4GALT* transcripts. NAT: untransfected 2102Ep cells; WT: consensus Gb3/CD77 synthase; p. Q211E: Gb3/CD77 synthase with E at position 211; p. Q211D: Gb3/CD77 synthase with D at position 211; p. Q211N: Gb3/CD77 synthase with N at position 211

**Fig. 6:**
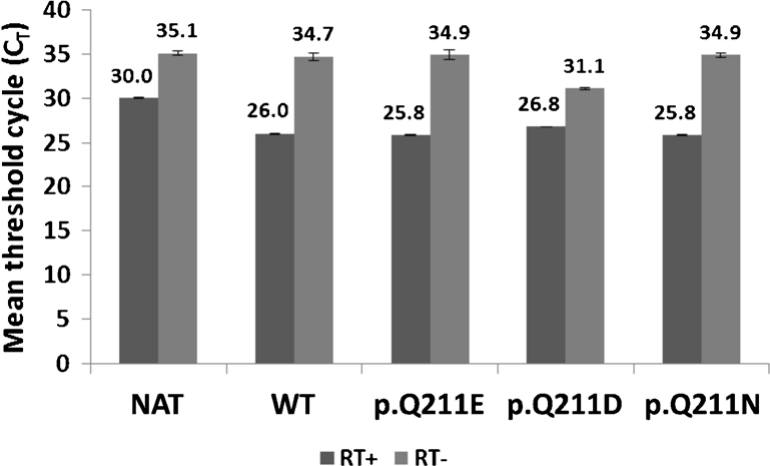
Comparison of mean threshold cycle (CT) values between RNA samples treated with reverse transcriptase (RT+) and untreated (RT-). NAT: untransfected 2102Ep cells; WT: consensus Gb3/CD77 synthase; p. Q211E: Gb3/CD77 synthase with E at position 211; p. Q211D: Gb3/CD77 synthase with D at position 211; p. Q211N: Gb3/CD77 synthase with N at position 211

Since the expression of P^k^, P1 and NOR antigens may be influenced by the mRNA level, we carried out a quantitative analysis of *A4GALT* transcripts in cells transfected with vectors encoding various forms of Gb3/CD77 synthase. The transcripts were markedly upregulated in all transfected cells (Fig. 5). The differences in endogenous transcript levels between clones were negligible. Comparison of the samples treated with reverse transcriptase (RT+) and untreated (RT-) shows that the differences in relative quantity (RQ) cannot be attributed to contamination with genomic DNA (Fig. 6).

## Discussion

The Gb3/CD77 synthase is an unusual glycosyltransferase, because it can transfer galactose to two different oligosaccharide acceptors: lactosylceramide and paragloboside, both of which contain terminal galactose [27]. When E instead of Q is present at position 211, the enzyme transfers galactose also to globoside, which contains terminal *N*-acetylgalactosamine. This makes the enzyme even more unusual, because glycosyltransferase changing acceptor specificity due to a point mutation had not been described until year 2012, when we showed that such mutation is the culprit behind NOR antigen synthesis [8]. Thus, the amino acid residue at position 211 seems to play an important role in the enzyme specificity. Since relatively little is known about the mechanism of Gb3/CD77 synthase action, and the crystal structure has not been solved yet, we embarked on the site-directed mutagenesis study combined with quantitative analysis of the expressed antigen. We substituted the consensus glutamine with either asparagine or aspartic acid, transfected the 2102Ep cells with vectors encoding such constructs, established stable clones, and evaluated the cells by flow cytometry, high-performance thin-layer chromatography and real-time PCR. Asparagine and aspartic acid have been selected, because they are structurally the closest counterparts to glutamine and glutamic acid, respectively, differing by only one methylene group (−CH2-) in the side-chain (one group in Asn and Asp, versus two groups in Gln and Glu). We found that cells expressing enzymes with D and N at position 211 completely lost the ability to produce P1 antigen and did not synthesize NOR antigen, while synthesis of P^k^ antigen remained at the constitutive level. However, as described previously, the Gb3/CD77 synthase with p. Q211E substitution acquired the ability to produce NOR antigens and at the same time the anti-P1 antibody binding capacity was markedly decreased in comparison to the cells transfected with the consensus enzyme. Real-time PCR analysis showed that the *A4GALT* mRNA levels in cells transfected with vectors were about 25–30 times higher than the levels of mRNA in the untransfected cells (which were derived only from the endogeneous *A4GALT* transcription). In addition, the mRNA level of Gb3/CD77 synthase with p. Q211E substitution was 45 % higher than the level of consensus enzyme mRNA. Since mouse anti-P1 antibody binding capacity of the cells expressing the enzyme with p. Q211E substitution was 1.4 times lower than in the case of cells expressing the consensus enzyme (WT), it may be speculated that the P1-synthesizing activity of the enzyme with substitution of Q by E is about 3 times lower than the activity of the consensus enzyme. Enzymes with other substitutions (D and N) do not reveal any activity at all. Thus, p. Q211E substitution causes a marked decrease of the enzyme activity toward one of its original acceptors (paragloboside), while conferring activity toward new acceptor, Gb4Cer, which gives rise to NOR antigen. Basing on these results, we hypothesize that activity of Gb3/CD77 synthase depends heavily on the presence of glutamine (preferred amino acid) or glutamic acid (less favorable) at position 211. Since neither asparagine nor aspartic acid can replace these residues without abolishing the enzyme activity, it may be speculated that none of the remaining proteinogenic amino acids would allow preservation of its catalytic properties. In addition, since N and D differ from Q and E by only one methylene group, respectively, our results suggest that size of the 211 residue side chain is crucially important for activity and apparently more important than the type of functional group in the side chain. These observations raise the possibility that the amino acid residue at position 211 of Gb3/CD77 synthase may be located near the active center of the enzyme, which requires further studies. Finally, since it seems that NOR antigens represent a minor fraction of the Gb3/CD77 synthase products (about 2.5 % of P1 specific binding capacity), it may be argued that NOR-synthesizing pathway is a small offshoot of enzyme activity.

When analyzing influence of amino acid replacement on glycosyltransferase activity, one must take into consideration that such change may affect intracellular localization of the enzyme. However, in our opinion it is highly unlikely, because glutamine, asparagine, glutamic acid and glutamine are very similar amino acids, differing only by either amido group or methylene group. Furthermore, 211 amino acid position of Gb3/CD77 synthase is substantially distant from the predicted transmembrane domain, which is the primary region contributing to subcellular localization of glycosyltransferases. In addition, precise determination of intracellular localization of the enzyme would require tagging (*e.g.* with myc-tag or FLAG-tag), which may impede protein folding and/or influence interaction with other proteins. Such studies will be more feasible and less fallible once a specific anti-enzyme antibody is available.

Indisputably, substitution of one amino acid residue in the glycosyltransferase can alter, broaden or abolish its activity and consequently lead to changes in the synthesized blood antigens. Several papers were published on changes in donor specificity of different glycosyltransferases. The most thoroughly studied glycosyltransferases are GTA (α1,3-*N*-acetylgalactosaminyltransferase) and GTB (α1,3-galactosyltransferase), the enzymes synthesizing ABO blood group antigens. They catalyze the transfer of GalNAc or Gal, respectively, from a UDP-sugar donor to Fucα1-2Galβ-R acceptor substrates. GTA and GTB are highly homologous enzymes, encoded by the same locus and differing at only four positions of the 354-amino acid polypeptide [28]. It was shown that a change of only one of these four residues renders the mutant enzyme able to transfer either of the two sugar residues, galactose and *N*-acetylgalactosamine, from UDP-Gal and UDP-GalNAc, respectively, and leads to the *cis*-AB phenotype [6]. Two amino acid residues, 266 and 268, are critical for altering the specificity of transferases A and B. Mutations c.796C > A (p. L266 M) and c.803G > C (p. G268 A) cause enzyme to transfer galactose instead of *N*-acetylgalactosamine [29]. GTB cannot bind UDP-GalNAc, since the active site comprises amino acid residues with longer side chains (266 M and 268 A), which restrict access to a larger substrate. In the case of GTA, smaller amino acid residues (268G and 266 L) allow the binding of *N*-acetylgalactosamine [2]. It was recently shown that the glycine residue present at position 266 in *cis*-AB transferase allows both nucleotide sugars to enter the active site of the enzyme [30].

Another example of how single amino acid residue can modulate enzyme activity is β1,4-galactosyltransferase, an enzyme that normally transfers Gal from UDP-Gal to GlcNAc residue. Replacement of Y at position 289 to L, I or N broadens the donor specificity, so mutated enzyme can use both UDP-Gal and UDP-GalNAc as the donor [7].

α1,3/4-fucosyltransferases encoded by *FUT3* (responsible for synthesis of Lewis histo-blood group antigens) and *FUT5*, are able to transfer fucose residues to the penultimate *N*-acetylglucosamine of two different acceptors, called type 1 and type 2, while most of their closest counterparts (α1,3-fucosyltransferases) employ only type 2 substrate. Both acceptors are terminated with galactose residue, but it is β1,4-linked and β1,3-linked to *N*-acetylglucosamine in the case of type 2 and type 1, respectively. Thus, the bispecificity is dictated by structure of the two substrates (each presenting only one free position for fucose attachment, 3 or 4, with the other being already occupied by the terminal galactose), rather than preference of the enzymes. However, the bispecificity depends on only one amino acid residue: *FUT3* and *FUT5* fucosyltransferases contain W at position 111, which is occupied by R in all monospecific α1,3-fucosyltransferases [31]. What still makes Gb3/CD77 synthase unique is that it can transfer the same sugar not just to different acceptors that differ *en bloc*, but in addition the glycosidic bond may be formed with two different sugar residues (Gal and GalNAc) that cap these acceptors, a phenomenon never described before in relation to a single point mutation [8]. In conclusion, we show that a single amino acid change has a dramatic effect on the specificity and activity of Gb3/CD77 synthase. We confirm that replacement of Q with E at position 211 of Gb3/CD77 synthase results in the formation of P^k^, P1 and NOR antigens and for the first time evaluate quantitatively the ability of the enzyme to synthesize different products. However, our data show that substitution of Q or E at this position by similar amino acids causes a complete abolition of the enzyme activity, which suggests that the amino acid 211 is crucially important for Gb3/CD77 synthase activity and may be directly involved in the catalytic reaction. The structure and kinetics of the consensus and the p. Q211E form of Gb3/CD77 synthase are currently under investigation.
